# Rhizospheric bacteria from the Atacama Desert hyper-arid core: cultured community dynamics and plant growth promotion

**DOI:** 10.1128/spectrum.00056-24

**Published:** 2024-04-30

**Authors:** Juan Castro-Severyn, Jonathan Fortt, Mariela Sierralta, Paola Alegria, Gabriel Donoso, Alessandra Choque, Andrea M. Avellaneda, Coral Pardo-Esté, Claudia P. Saavedra, Alexandra Stoll, Francisco Remonsellez

**Affiliations:** 1 Laboratorio de Microbiología Aplicada y Extremófilos, Departamento de Ingeniería Química y de Medio Ambiente, Facultad de Ingeniería y Ciencias Geológicas, Universidad Católica del Norte, Antofagasta, Chile; 2 Centro de Investigación Tecnológica del Agua y Sustentabilidad en el Desierto-CEITSAZA, Universidad Católica del Norte, Antofagasta, Chile; 3 Laboratorio de Ecología Molecular y Microbiología Aplicada, Departamento de Ciencias Farmacéuticas, Facultad de Ciencias, Universidad Católica del Norte, Antofagasta, Chile; 4 Laboratorio de Microbiología Molecular, Departamento de Ciencias Biológicas, Facultad de Ciencias de la Vida, Universidad Andres Bello, Santiago, Chile; 5 Laboratorio de Microbiología Aplicada, Centro de Estudios Avanzados en Zonas Áridas CEAZA, La Serena, Chile; 6 Instituto de Investigación Multidisciplinar en Ciencia y Tecnología, Universidad de la Serena, La Serena, Chile; Instituto de Ecología, A.C. (INECOL), Pátzcuaro, Mexico

**Keywords:** *D. spicata*, *S. foliosa*, soil cultures, rhizobiome, amplicon sequencing

## Abstract

The Atacama Desert is the oldest and driest desert on Earth, encompassing great temperature variations, high ultraviolet radiation, drought, and high salinity, making it ideal for studying the limits of life and resistance strategies. It is also known for harboring a great biodiversity of adapted life forms. While desertification is increasing as a result of climate change and human activities, it is necessary to optimize soil and water usage, where stress-resistant crops are possible solutions. As many studies have revealed the great impact of the rhizobiome on plant growth efficiency and resistance to abiotic stress, we set up to explore the rhizospheric soils of *Suaeda foliosa* and *Distichlis spicata* desert plants. By culturing these soils and using 16S rRNA amplicon sequencing, we address community taxonomy composition dynamics, stability through time, and the ability to promote lettuce plant growth. The rhizospheric soil communities were dominated by the families Pseudomonadaceae, Bacillaceae, and Planococcaceae for *S. foliosa* and Porphyromonadaceae and Haloferacaceae for *D. spicata*. Nonetheless, the cultures were completely dominated by the Enterobacteriaceae family (up to 98%). Effectively, lettuce plants supplemented with the cultures showed greater size and biomass accumulation. We identified 12 candidates that could be responsible for these outcomes, of which 5 (*Enterococcus, Pseudomonas, Klebsiella, Paenisporosarcina,* and *Ammoniphilus*) were part of the built co-occurrence network. We aim to contribute to the efforts to characterize the microbial communities as key for the plant’s survival in extreme environments and as a possible source of consortia with plant growth promotion traits aimed at agricultural applications.

**IMPORTANCE:**

The current scenario of climate change and desertification represents a series of incoming challenges for all living organisms. As the human population grows rapidly, so does the rising demand for food and natural resources; thus, it is necessary to make agriculture more efficient by optimizing soil and water usage, thus ensuring future food supplies. Particularly, the Atacama Desert (northern Chile) is considered the most arid place on Earth as a consequence of geological and climatic characteristics, such as the naturally low precipitation patterns and high temperatures, which makes it an ideal place to carry out research that seeks to aid agriculture in future conditions that are predicted to resemble these scenarios. Our main interest lies in utilizing microorganism consortia from plants thriving under extreme conditions, aiming to promote plant growth, improve crops, and render “unsuitable” soils farmable.

## INTRODUCTION

The Atacama Desert is in the dry subtropical climate belt between 18°S and 27°S, extending from the coastal edge to the Andean Mountain complex, and is the oldest and driest desert on Earth (1, 2). This ecosystem is considered a natural laboratory due to its environmental co-occurring conditions, including high variations in temperature, ultraviolet (UV) radiation, hydric stress, and the presence of metal(oids), among many others (3). Also, soil weathering, leaching, and water erosion rates are slow in the area (4, 5). Despite hostile conditions, the Atacama Desert harbors vast adapted life forms making it possible to find important biodiversity. Among these, hypolithic cyanobacteria (6), non-lichenized fungi (7), lichens (8), cacti (9), and even shrubs and trees (10) have been reported. Moreover, the Atacama Desert hyper-arid core is a hotspot for studies on astrobiology and polyextremophile life (11–13). This ecosystem is under constant hydric stress, as the annual mean precipitation is lower than 2 mm, and there are years that do not receive any rain (14), making this area an ideal model for understanding the basis of abiotic stress resistance.

Highly adapted microbial taxa thrive in these hyper-arid environments by having multiple adaptations for effective colonization and stress tolerance (15, 16). Particularly, members of the Firmicutes, Bacteroidota, and Actinobacteria phyla (Rubrobacterales, Actinomycetales, and Acidimicrobiales) have been associated with halite nodules and soil samples from these environments, as they can develop with low humidity, high soil salinity, and high solar radiation conditions (14, 17–19). Moreover, increases in soil organic matter levels associated with the presence of vegetation have been described as vital for these types of extreme environments (20, 21 ), as well as symbiotic interactions between microorganisms and plant roots, which contribute to soil nutrient recycling (7, 22–24). Also, plants form patches of vegetation that generate spatial heterogeneity and changes in pH at different scales in the soil due to moisture and nutrient retention (25, 26). These plant-microbe symbiotic relationships are further promoted in extreme environments as they can increase the species’ survival under stress conditions, independently of their individual characteristics (27, 28). Some microorganism traits from which plants can benefit are salt tolerance, zinc, potassium, and phosphorus solubilization, ammonia siderophores, phytohormones, and secondary metabolite production (29). Nonetheless, the key groups and the role they would be playing in the interaction under these particular extreme environments remain unclear.

The use of microorganisms that contribute beneficial traits for plant development and increased yield can be a successful strategy to aid current agricultural challenges in the face of climate change and desertification (30–32). Moreover, strains isolated from plant species living in highly challenging and stressful environments have been evaluated for their protective capacity against stress conditions or/and growth promotion in crops (33, 34). The capacity of some microorganisms to induce drought resistance, improve photosynthetic rates, promote phytohormones, and increase biomass (35–38) has been tested in *Lactuca sativa, Chenopodium quinoa,* and *Hordeum vulgare* crops*,* which are relevant for northern-Chilean agriculture and also highly demanded foods for human consumption worldwide (39–43). Despite the fact that most of these investigations are based on isolated bacteria or yeast strains, there is compelling evidence fostering the use of microbial consortia as biofertilizers to promote growth in challenging conditions, increase crop yield, nutrient uptake, and salinity stress (30, 44–48). However, there are many technical aspects that hinder the use and application of complex consortia as biofertilizers, where the greatest difficulty lies in their maintenance over time, reproducibility, and legal regulation (49, 50). Despite this, no work has yet been done addressing the taxonomic composition of complex consortia cultured from environmental samples that are monitored over time.

Our group identified an oasis in the Yungay area of the Atacama Desert hyper-arid core (The Aguas Blancas Basin). This place is uncoupled from the coastal fog due to a mountain range, and rainfall is currently insufficient to support vascular plants in most of the area (51). Nonetheless, a small oasis can be found harboring a high density of plants and shrubs that thrive facing the high salinity soils, extreme drought, and UV radiation (10). The most abundant plant is *Distichlis spicata,* which is well adapted to grow in alkaline saline soils and also contributes to the construction of mounds around its individuals, favoring localized elimination of salts by capillary action and evaporation. *Suaeda foliosa* is a perennial decumbent plant that does not require large amounts of water for development since it is specialized in capturing and retaining it (52–54). In this study, we set up to characterize the microbial community composition of *S. foliosa* and *D. spicata* rhizospheric soils, then culture these soils to monitor the taxonomic dynamic and stability through time and several subcultures to finally test the ability of these cultured consortia to promote *L. sativa* growth, identifying which taxa could be playing a key role in the lettuce improvement.

## MATERIALS AND METHODS

### Field trip and sample collection

**Fig 1:**
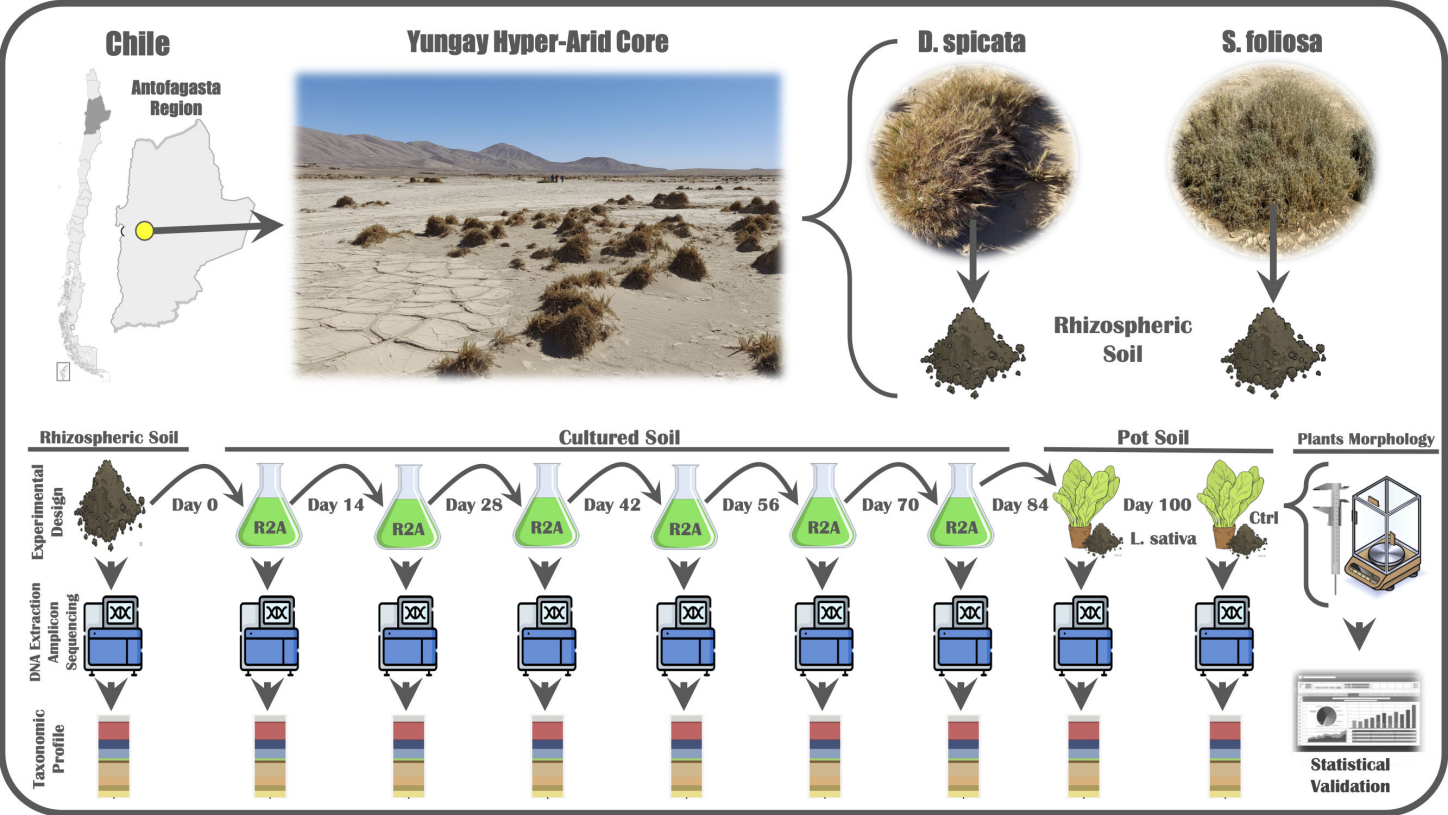
Sampling and experimental design scheme. In the upper panel, we can see the location of the Yungay Hyper-Arid Core (22°S and 26°S) and the fertile island (24°3′29.93″S and 69° 49′33.25″O) and the sampled plant species photos. In the lower panel, we can see the experimental design following the rhizospheric soil subcultures through time and the points at which DNA was extracted to determine the taxonomic composition. Map modified from Wikimedia Commons user B1mbo under CC BY-SA license.

A sampling expedition was carried out in September 2022 to the Yungay Hyper-Arid Core area, located in the Antofagasta Region of Chile [between approximately 22°S and 26°S (14, 22)]. The mean annual rainfall for this area is around 2.0 mm (making it the driest zone in the desert), and long-term climate data indicate that it is also the driest non-polar desert on Earth, due to low rainfall, high temperatures, and evapotranspiration rates, as well as the prevalent cloudless condition, low total ozone column, the highest surface ultraviolet radiation, and total solar irradiance recorded in the planet (55, 56) (3). Despite all these conditions, we located a small oasis area or fertile island (24°3′29.93″S and 69°49′33.25″O; Fig. 1, upper panel) with the presence of three plants species: *Distichlis spicata* is a grass (the most abundant one), also known as “desert saltgrass”; *Suaeda foliosa* is a bush (representing much lower coverage); and finally, one specimen of *Prosopis tamarugo,* which is a leguminous deciduous tree. Rhizospheric soil samples were taken from *D. spicata* and *S. foliosa* in an aleatory sampling, trying to cover as much of the area as possible (three sampling points with three specimens for each plant). We dug with a (70% ethanol sterilized) hand shovel, the closest to the plant root possible (in a 5 cm radius from the plant’s main root and 10 cm of depth) without affecting it. Soil samples were collected in 50 mL sterile falcon tubes and immediately transported to the laboratory.

### Soil cultures

Approximately 0.5 g of rhizospheric soil was inoculated into flasks with 20 mL of sterile Reasoner’s 2A (R2A) medium [(57), 0.5 g/L yeast extract, 0.5 g/L proteose peptone, 0.5 g/L casamino acids, 0.5 g/L dextrose, 0.5 g/L soluble starch, 0.5 g/L sodium pyruvate, 0.3 g/L dipotassium phosphate, and 0.03 g/L magnesium sulfate, pH 7.2] and cultured at 20°C with constant agitation (120 rpm) in triplicates for each plant species. After 14 days, 1 mL of the grown culture was inoculated in a new flask containing 20 mL of fresh sterile R2A medium to continue culturing for 14 more days under the same conditions (Fig. 1, lower panel); the remaining culture was centrifuged (3,000 *g*, 10 min) to discard the supernatant, and the pellets were stored at −80°C until DNA extraction. This process was repeated four more times until reaching day 84, the day on which the cultures were divided into three fractions. The first fraction was pelleted and stored at −80°C until DNA extraction (the remaining two parts were used for the potted plant experiments). The culture’s growth was monitored weekly through the OD_600_ reading.

### Potted plant experiment

For this experiment, 15-day-old green leaf lettuce (*Lactuca sativa* var. crispa) seedlings were obtained from Vivero La Portada (Antofagasta, Chile). The seedling roots were carefully washed with sterile distilled water and then soaked in a culture suspension for 1 h. This suspension was prepared for both plant species by pooling the replicates of the day 84 cultures (second fractions) and diluting at a 1:10 ratio with sterile distilled water. Then, soaked and control (non-soaked) seedlings were transplanted to 250 mL pots with a sterilized substrate mixture (2:1:1, leaf mold/sand/perlite) and grown for 16 days in an open-air shadehouse with a natural photoperiod of 12 h/12 h, approx. This was carried out under the climatic conditions of Antofagasta city, with extreme UV radiation (>11), 17.5°C–22.5°C of average annual temperature, 0 mm of precipitation, ~72.8% of air humidity, and 18.52–27.72 km/h winds (according to the Chilean Meteorological Direction 2022 report). Moreover, four treatments were tested: *L. sativa* plants inoculated with *D. spicata* culture; *L. sativa* plants inoculated with *S. foliosa* culture; *L. sativa* plants without any inoculation (Control), and *L. sativa* plants supplemented with 1:10 diluted R2A sterile media (ControlMed). Each treatment consisted of 12 independent and randomized replicates (plants). Regular irrigation was carried out every 48 h with 50 mL of (non-sterile) distilled water in the afternoon. On day 8 after transplant, a second inoculation with 50 mL of culture suspension (prepared by pooling the replicates of the day 84 cultures’ third fractions) was carried out in the corresponding pots. Afterward, normal irrigation with water was continued in the same way until day 100, when the lettuce plants were harvested and corresponding rhizosphere soil samples were taken from four selected pots (for each treatment) and stored at −80°C until DNA extraction.

### Plants’ morphological evaluation

After the harvest, plant roots were carefully washed with distilled water to remove any remaining substrate and then they were air-dried over paper towels for 30 min. Later, plants were weighed (fresh weight), and the longest root and the second “true leaf” lengths were measured for each plant. Next, each plant was deposited inside a paper bag, and these were dried in a laboratory stove at 70°C for 48 h and then weighed again (dry weight) to calculate the dry matter content. All data were carefully recorded, and the statistical significance was tested through one-way ANOVA with *post hoc* Tukey’s HSD for all comparisons (GraphPad 5.0 Prism), and visualizations were made using the R package ggplot2 (58).

### DNA extraction and amplicon sequencing

Total DNA was extracted from *S. foliosa* and *D. spicata* rhizospheric soil, culture pellets, and potted plant experiment soils using the E.Z.N.A. Soil DNA Extraction Kit (Omega Bio-tek, USA) according to the manufacturer’s instructions. DNA integrity, quality, and quantity were verified by 1% agarose gel electrophoresis, OD_260/280_ ratio spectroscopy, and fluorescence using a Qubit 3.0 fluorometer along with the Qubit dsDNA HS assay kit (Thermo Fisher Scientific, USA). Next, DNA samples were sent to the Environmental Sample Preparation and Sequencing Facility at the Argonne National Laboratory (IL, USA) for the amplification of the bacterial 16S rRNA gene V4 region (~250 bp) using the 515F and 806R primers (59) and construction of 151 bp paired-end libraries and sequencing on a MiSeq (Illumina) platform.

### Taxonomic composition and diversity analysis

This analysis was conducted in R v4.2.2 and RStudio v1.3.1093 following the DADA2 v1.26.0 R package pipeline (60) in order to infer amplicon sequence variants (ASVs) present in each sample. Briefly, the reads were evaluated for quality control and subsequently trimmed (Ns = 0, length ≥ 130 bp, and expected errors ≤ 2), followed by dereplication, denoising, and merging of paired reads. Subsequently, the ASVs’ table was built with 97% clustering, the chimeras were removed, and taxonomic assignment was carried out against the Silva v138 (61) database with the Ribosomal Database Project’s naive Bayesian classifier (62). ASVs identified as Eukarya, Chloroplast, and Mitochondria were removed. Moreover, a multi-sequence alignment was created with DECIPHER v2.26.0 (63) to infer phylogeny using FastTree v2.1.11 (64). Furthermore, a phyloseq-object (containing the ASVs, taxonomy assignment, phylogenetic tree, and the sample meta-data) was created using the R package Phyloseq v1.42.0 (65). Replicates per condition were averaged by day or stage. The ASV counts were normalized by variance-stabilizing transformation using the R package DESeq2 v1.38.3 (66). Alpha diversity indices were calculated using the Microbiome v1.20.0 and Btools v0.0.1 packages. Plots were generated using the ggpubr v0.6.0 package with comparisons between plant species using the Wilcoxon test (*P* < 0.05), and the statistical significance of the community variation throughout the experiments was evaluated with ANOVA and the Kruskal-Wallis test. Taxonomy composition and relative abundance plots were generated using the ggplot2 v3.4.1, fantaxtic v0.2.0, and ampvis2 v2.7.35 (67) R packages. Moreover, candidate taxa were identified by filtering with the following criteria to keep those that (i) were detected in the rhizospheric soils of Yungay; (ii) were detected (maintained) throughout the 84-day cultures; (iii) were detected in the pot soil (after the bio-fertilization experiments of *L. sativa*); and (iv) had a higher abundance in the bio-fertilized pot soil, compared to the control pot soil. Finally, co-occurrence networks were constructed by agglomerating the phyloseq object at best hit using the microbiomeutilities v1.00.11 R package (68). The network was estimated using the SpiecEasi v0.1.4 (69) R package (neighborhood selection model) and visualized with GGally v1.5.0 (70) R package.

## RESULTS

The direct inoculation of rhizospheric soil from Yungay into the culture medium effectively resulted in the propagation of microorganisms. This microbial growth was evident with increased medium turbidity, and although there was growth in all the subcultures, the DO_600_ monitoring every 14 days revealed a negative slope when comparing the cultures of both plant species throughout time (Fig. S1). Initially, on day 14, the cultures from both plants displayed very different values (being higher than those from *D. spicata*). This difference progressively decreased as the growth rate of both cultures became slower, and from day 56, they became equivalent.

**Fig 2:**
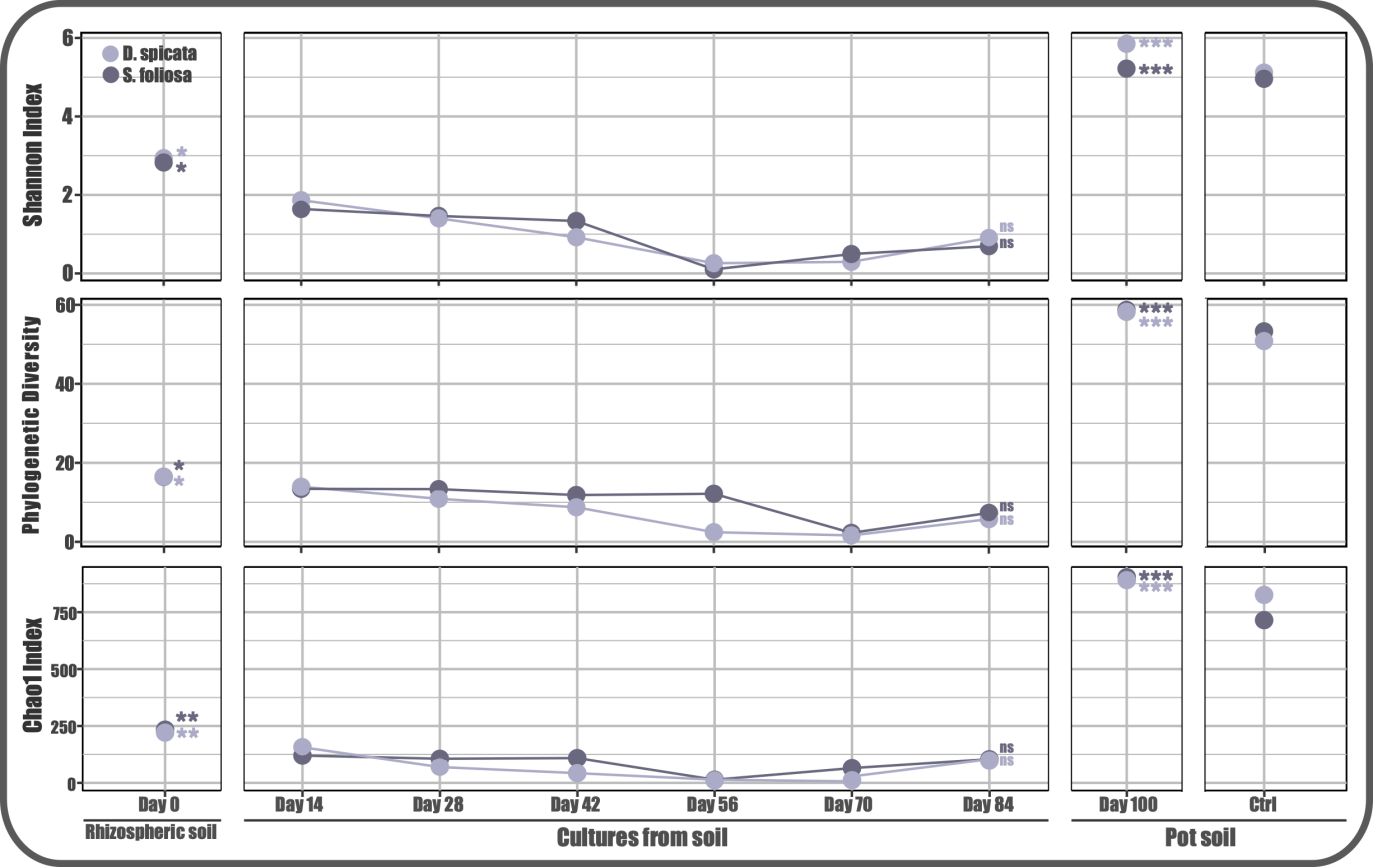
Alpha diversity monitoring through time and experimental stages. Faith’s phylogenetic diversity, Chao1, and Shannon indices were calculated for each evaluated point, and their average values are shown in red for *S. foliosa* experiments and in black for *D. spicata* (*P > 0.05, **P > 0.01, ***P > 0.001 and ns: not significant).

Alpha diversity metrics were monitored throughout the experiment and compared between the stages and as expected, all evaluated indices decreased throughout the experiment (Fig. 2). No significant differences were observed between both plants in the three used metrics, even when considering only the rhizospheric soil communities of *S. foliosa* and *D. spicata* (Fig. S2). Furthermore, changes among the stages or through time are evident in the three indices. The Shannon diversity index decreased progressively in cultures of microorganisms obtained from the Yungay rhizospheric soils toward day 56 culture; subsequently, they remained roughly stable until day 84, reaching the highest value on the lettuce pot soils. Moreover, the same pattern is observed for the Chao1 index and the phylogenetic diversity but to a lesser extent. In addition, the soil from the control lettuce pots displayed similar values for the three metrics to the inoculated ones.

**Fig 3:**
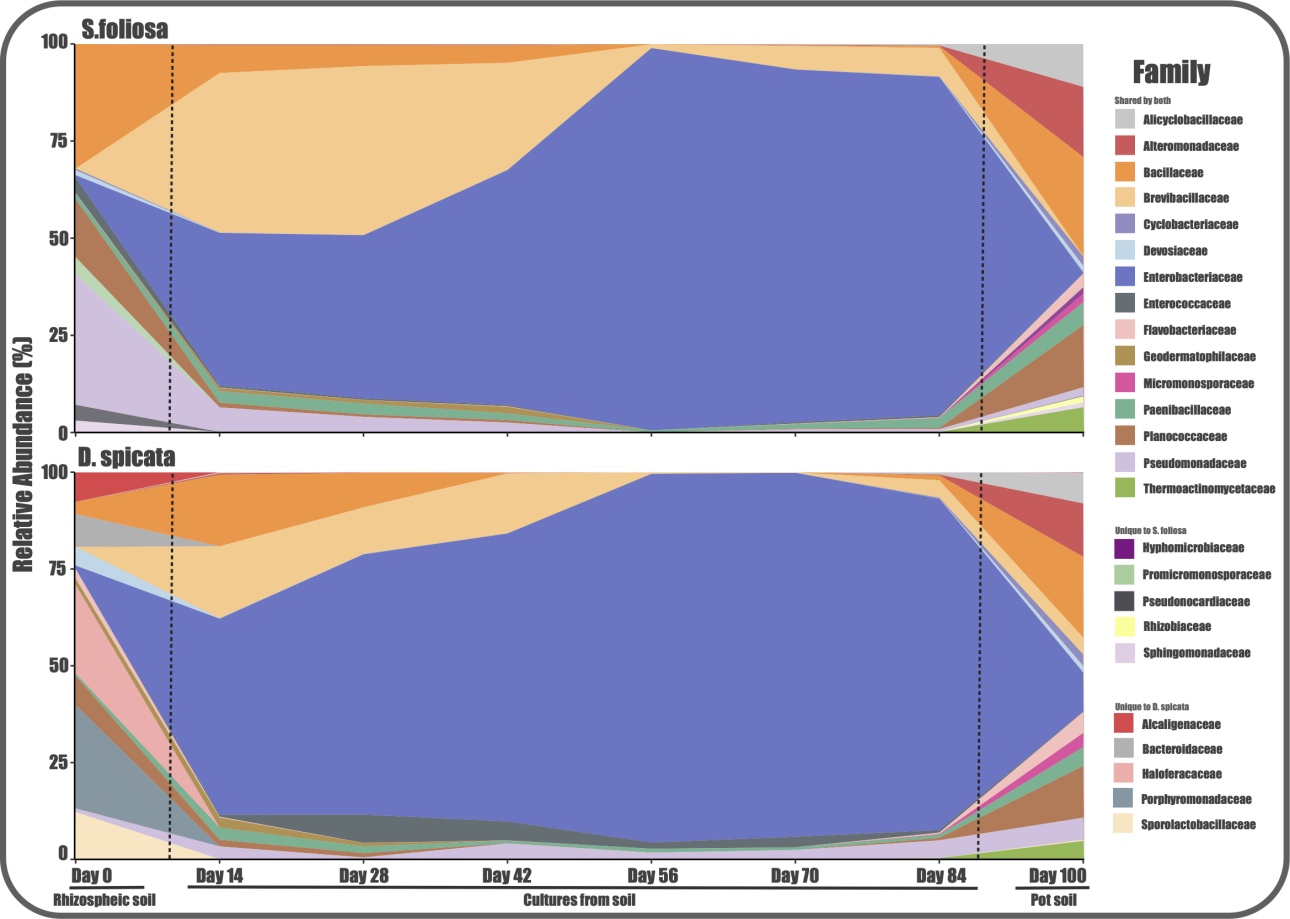
Taxonomic composition dynamics through time and experimental stages. Taxa were agglomerated to display the relative abundance of the top 20 family ranks for both plant species (code by colors).

By analyzing the taxonomic composition throughout all stages of the experiment, we were able to identify a total of 1,017 ASVs classified to the lowest taxonomic rank available (99.8% at phylum, 99.02% at class, 92.6% at order, 82.9% at family, 58.01% at genus, and 8.16% species). The monitoring of taxonomic groups evidenced that over time some populations were selected and progressively enriched in the cultures, which is very clear within the Enterobacteriaceae family in both plants (Fig. 3), which reached 95.2% and 98.4% of relative abundance by day 56 for *D. spicata* and *S. foliosa* cultures, respectively. This reflects the aforementioned decrease in diversity throughout time observed for the cultures of both plants. The Brevibacillaceae family is also enriched in the culture stage, reaching 18.6% and 43.5%, respectively, for both plants. On the other hand, families such as Bacillaceae (25.4%) and Pseudomonadaceae (6.3%), which were the main representatives of *S. foliosa* rhizospheric soils, were depleted throughout the culture period. Similarly, the Haloferacaceae (22.9%) and Porphyromonadaceae (26.7%) families disappear during *D. spicata* cultures.

Moreover, data show that communities become more homogeneous during the culture stage, regardless of the soil sample origin; there were some differences among the detected families. The Hyphomicrobiaceae, Rhizobiaceae, Sphingomonadaceae, Promicromonosporaceae, and Pseudonocardiaceae families were only detected in the *S. foliosa* rhizospheric soil, while the Porphyromonadaceae, Haloferacaceae, Sporolactobacillaceae, Alcaligenaceae, and Bacteroidaceae families were unique for *D. spicata* rhizospheric soil. Also, the most abundant or dominant taxa belonged to the Pseudomonadaceae, Bacillaceae, and Planococcaceae families for *S. foliosa* and the Porphyromonadaceae and Haloferacaceae families for *D. spicata*. All these results are reflected at the phylum rank with a significant presence and subsequent complete dominance by Firmicutes and Proteobacteria (Fig. S3).

**Fig 4:**
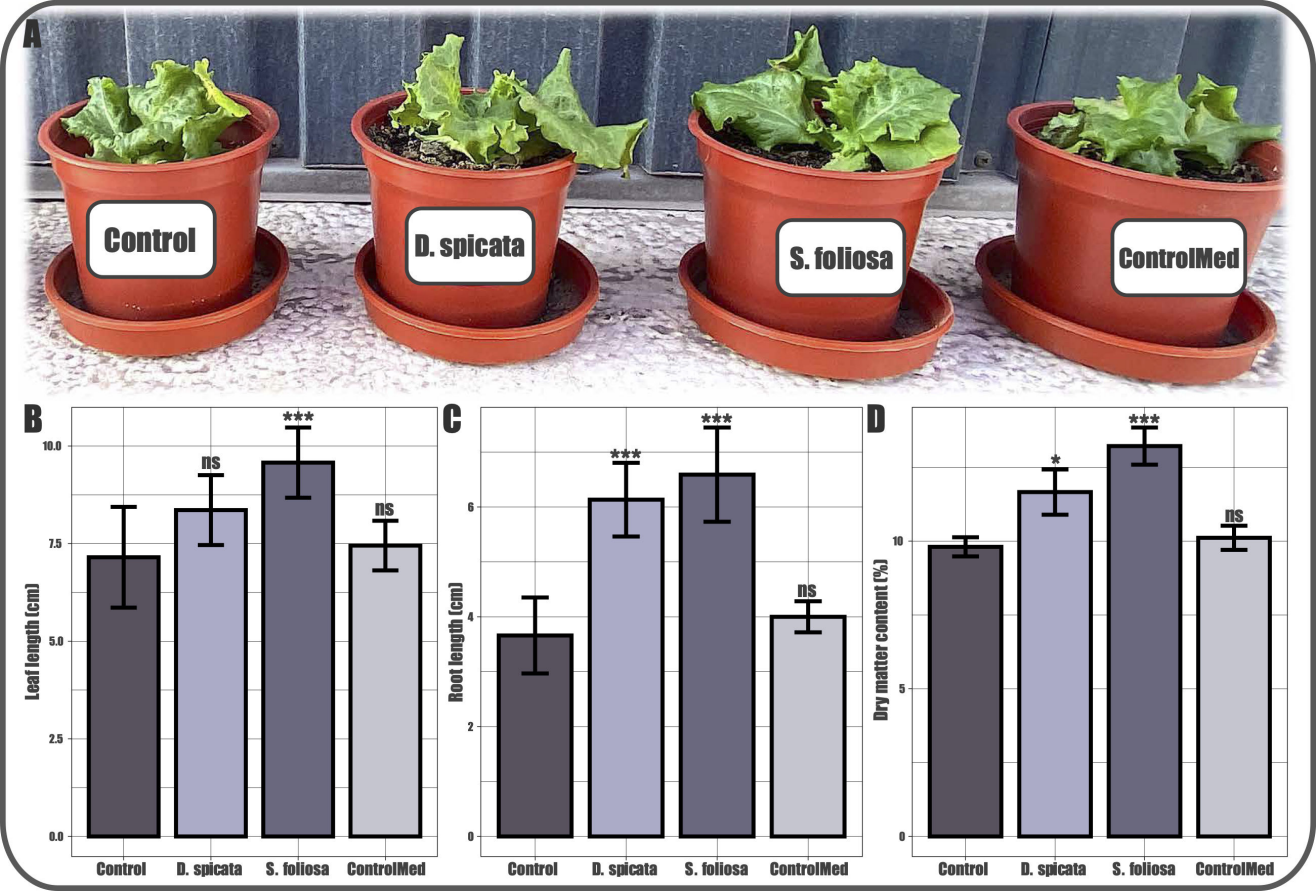
Effect of bacterial culture inoculation on *L. sativa* growth. (**A**) Picture of the day 100 lettuce plants in the four different treatments: control (not supplemented); *D. spicata* (supplemented with the day 84 culture from the *D. spicata* rhizospheric soil); *S. foliosa* (supplemented with the day 84 culture from the *S. foliosa* rhizospheric soil), and ControlMed (supplemented with sterile culture medium). (**B**) Leaf length in centimeters. (**C**) Root length in centimeters and (**D**) dry matter content in percentage. Presented data are an average of at least 10 individuals. *P ≤ 0.05; **P ≤ 0.01; and ***P ≤ 0.001 indicate statistical significance (regarding the control) according to ANOVA.

The inoculation of lettuce plants with the 84-day cultures of both *S. foliosa* and *D. spicata* rhizospheric bacteria resulted in growth promotion of the plant compared to those that were not inoculated with any type of culture or microorganisms. The supplemented ones were evidently larger and visually vigorous (Fig. 4A). These differences were morphologically quantified considering the root length (Fig. 4B), the leaf length (Fig. 4C), and the accumulated dry matter percentage (Fig. 4D). Notably, the lettuce plants that were inoculated with the cultures from *S. foliosa* rhizospheric soil showed a statistically significant increase in the three evaluated parameters. Alternatively, in those inoculated with the *D. spicata* cultures, the promotion was less evident; nonetheless, root length and dry matter content were significantly increased. Also, the lettuce plants inoculated with a sterile culture medium did not show significant differences in any of the parameters regarding the control plants.

As we are aiming to determine which could be the key agents that promote the growth of the lettuce plants inoculated with the cultures, we set up to identify candidate taxa. When evaluating the community composition agglomerated at genus rank, we observed mostly the enrichment of the *Klebsiella* and *Brevibacillus* genera as the culture progressed, while the composition patterns changed completely between the Yungay rhizospheric soils and the cultures, with an evident decrease in diversity as well. We also noticed that many of the detected families were represented by only one genus (Fig. S4). Moreover, other genera such as *Bacillus*, *Brevibacillus*, *Enterococcus,* and *Pseudomonas* were also detected in the cultures, although with a lower relative abundance. Also, comparing the community composition between both plants’ rhizospheric soil, we can highlight great differences such as the dominance of *Porphyromonas* and *Haloterrigena* in *D. spicata*, as well as the great abundance of *Pseudomonas* and *Bacillus* in *S. foliosa*.

**Fig 5:**
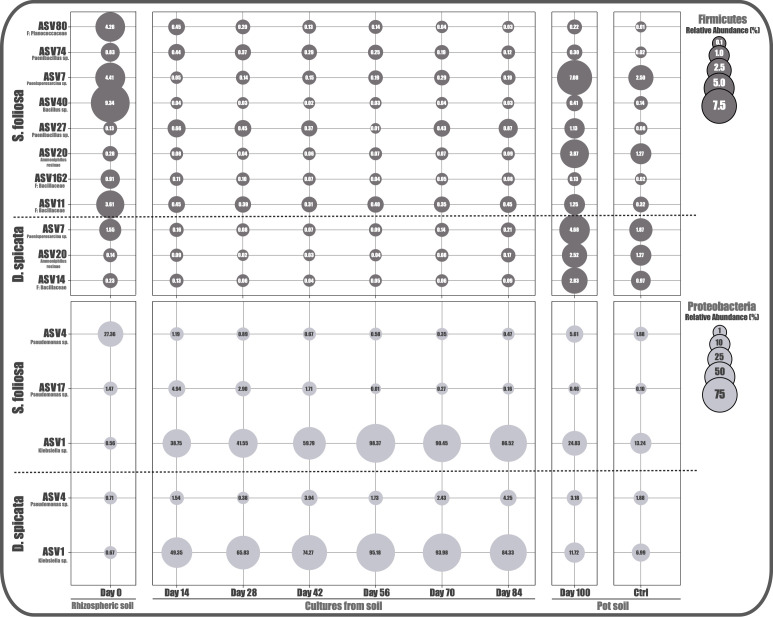
Potential key taxa identification. Taxa that meet the established criteria are shown in the bubble plot, where each circle represents the relative abundance of each taxon (best available rank classification) at the corresponding experimental stage. Legends are color-coded by phylum with the corresponding scale.

We identified 12 candidate ASVs that meet the selection criteria, of which three belong to the Proteobacteria phylum (ASV1: *Klebsiella* sp.; ASV4 and ASV17: two strains of *Pseudomonas* sp.) and nine to the Firmicutes phylum (ASV7: *Paenisporosarcina* sp.; ASV20: *Amorphotheca resinae*; ASV11: Enterococcus sp.; ASV40: *Bacillus* sp.; ASV27 and ASV74: two strains of *Paenibacillus* sp.; ASV80: a member of the Planococcaceae family; and ASV14 and ASV162: two members of the Bacillaceae family) (Fig. 5). Moreover, only 5 of the 12 ASVs were identified in the *D. spicata* experiments, while 11 of the 12 ASVs were identified in the *S. foliosa* experiments. Interestingly, one of the two Bacillaceae family members (ASV14) was exclusively detected on the *D. spicata* experiments, whereas ASV11, ASV17, ASV27, ASV40, ASV74, ASV80, and ASV162 were exclusively detected on the *S. foliosa* experiments. Additionally, 4 of the 12 candidate ASVs are shared between both plants (ASV1, ASV4, ASV7, and ASV20). Interestingly, *Pseudomonas* sp. (ASV4) and *Bacillus* sp. (ASV40), which represented an important proportion in Yungay rhizospheric soil communities, decreased their abundance greatly throughout the culture stages. On the contrary, *Klebsiella* sp. (ASV1), which represents less than 1% of relative abundance in the Yungay rhizospheric soil communities, was enriched throughout the cultures until reaching virtually total dominance (98.37%).

**Fig 6:**
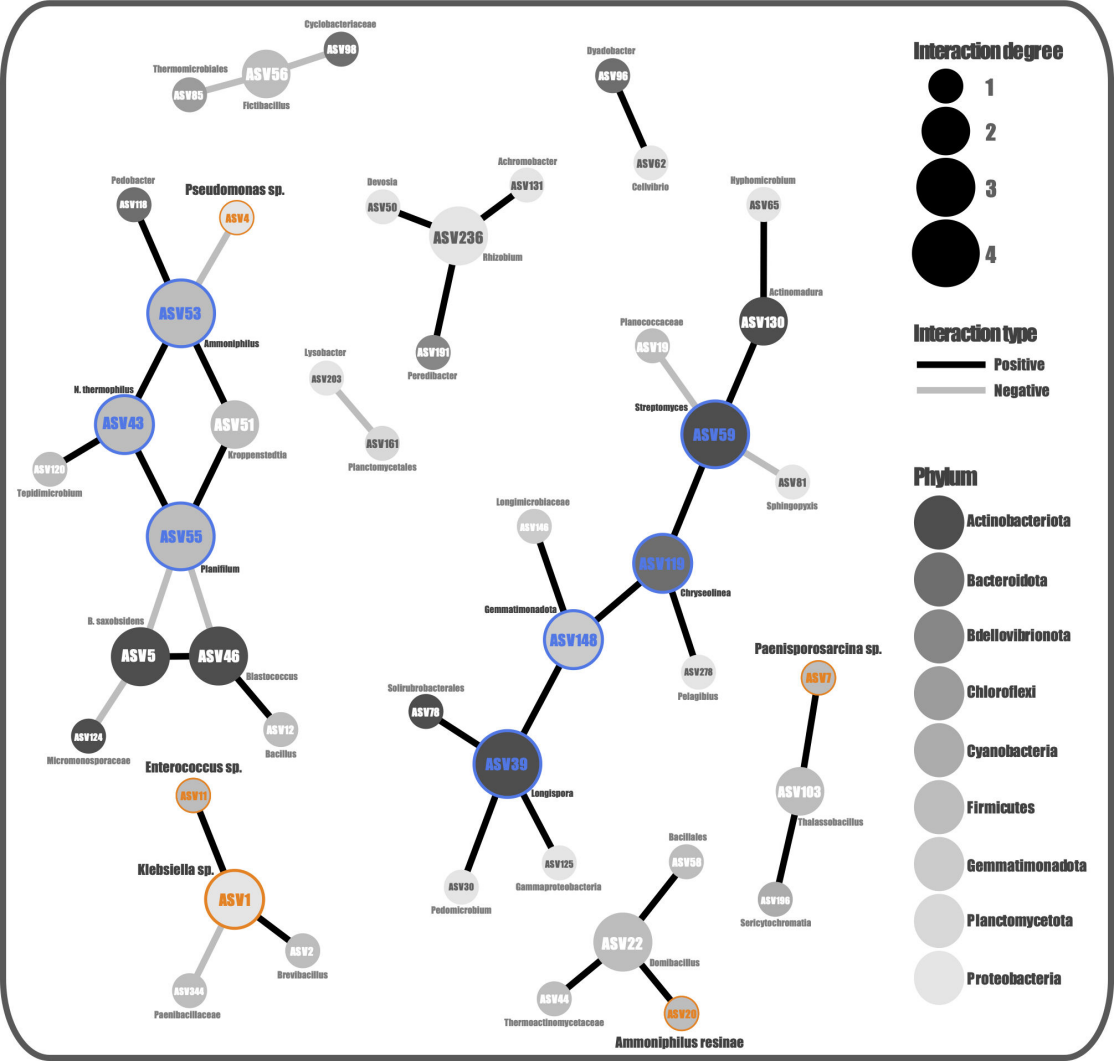
Co-occurrence networks of the *L. sativa* pot soil bacterial communities. The size of each node (representing ASVs) is proportional to the number of different interactions (degrees), the color of the edges (significant connection between nodes) represent the interaction type, the node color indicates the taxonomic affiliation at the phylum level, and node labels are at the lowest available taxonomic classification. The node border color denotes candidate species in orange and the identified keystone taxa in blue.

The co-occurrence network of the lettuce pot soil communities was composed of 46 nodes (ASVs with at least one significant correlation) and 39 edges arranged in nine modules (Fig. 6). The modules are dominated by Firmicutes (17/46) and Proteobacteria (12/46), agreeing with all previous findings. Also, the nodes with the highest degree of interaction belong to ASVs of Firmicutes and Actinobacteriota, despite this last one being least abundant in the communities. Moreover, 5 of the 12 identified candidates are part of the network structure (ASV1: *Klebsiella* sp., ASV4: *Pseudomonas* sp., ASV7: *Paenisporosarcina* sp., ASV11: Bacillaceae, and ASV20: *A. resinae*), which are mostly Firmicutes and belongs to four of the nine network modules. Particularly, ASV1 (*Klebsiella* sp.) is the central part of a module with four nodes, which may suggest an important structural role. Among its connections are ASV11 (*Enterococcus* sp.), which is another candidate, and ASV2 (*Brevibacillus* sp.), which was one of the most abundant genera in culture stages. Finally, for these pot soils, we identified seven keystone species (ASV39: *Longispora* sp., ASV43: *Novibacillus thermophilus*, ASV53: *Ammoniphilus* sp., ASV55: *Planifilum* sp., ASV59: *Streptomyces* sp., ASV119: *Chryseolinea* sp., and ASV148: Gemmatimonadota) mostly belonging to Firmicutes and Actinobacteria phyla, which might be playing an important role in the community function, structure, and stability.

## DISCUSSION

In this work, we were able to improve the growth of *L. sativa,* which is a relevant crop for Chilean agriculture due to its high consumer demand, using standard and affordable microbiology methods to culture bacterial consortia from the rhizosphere of plants thriving under extreme abiotic conditions of the Atacama Desert hyper-arid core. This is the first report where the culturable fraction of a rhizospheric soil community from this extreme environment was monitored, describing its dynamics throughout time (from a taxonomic composition point of view). The microbial cultures were maintained over six subcultures for a total period of 84 days, and as expected, the community diversity decreased. The composition was enriched with the best-adapted taxa to laboratory-controlled conditions, a critical aspect to consider for the development of biotechnologically applied tools, as the bacteria that are difficult to grow are not such attractive candidates for agricultural application.

The monitoring of the alpha diversity accounts for a selection process with a consequent and expected loss of diversity progressively. Although there were no significant differences between the cultures of both plant species, there was a decrease over time. It is worth noting that the taxonomic composition of *D. spicata* rhizospheric soil exhibited less variability compared to *S. foliosa*, which accounts for a more stable or selected community. This may be related to the plant’s need for more specialized organisms capable of tolerating the high salinity and pH changes induced in the soil, which are caused by the plant’s physiologic processes to eliminate salts and thus thrive under desert conditions (52, 53). We also must consider that the diversity of desert soils, although rich, is low compared to other types of samples such as sediments and even other rhizospheric soils (71, 72), particularly in the Yungay area of Atacama Desert, where there is an important diversity that is consistent with the extreme environmental conditions of the area (14, 73, 74). On the other hand, the higher diversity observed in the pot soils (after the inoculation experiments) could be mostly due to the lettuce’s own microbiome, which has been vastly described as having high diversity, and also to the plant exudates, which are known to affect microbial diversity dynamics in the rhizospheric soil (75, 76). Also, the irrigation water and the environment could be input sources, even though the substrate was sterilized prior to the experiments, which were carried out in an open environment since we tried to reproduce field conditions as much as possible.

The complete dominance or enrichment of *Klebsiella* genus and, to a lesser extent, the *Brevibacillus* genus reflects the selection process and accounts for what is seen at phylum (Proteobacteria and Firmicutes) and family (Enterobacteriaceae and Brevibacillaceae) ranks for both plants. Different species of the *Klebsiella* genus have been recurrently detected in many types of soils and environmental samples (77), even in the soils of the Yungay area (78). Regarding the association of *Klebsiella* with plants, there are many reports of the great repertoire of beneficial traits provided: ACC deaminase activity, atmospheric nitrogen fixation, inorganic phosphate solubilization, great adhesion capacity (to the plant roots), production of indole acetic acid, siderophores, cellulase, protease, and amylase enzymes, as well as the promotion of saline, drought, and oxidative stress tolerance, all of which have been demonstrated experimentally, including irrigation tests with seawater (79–83). Furthermore, the *Brevibacillus* genus has also been widely detected, isolated, and cultured from the tissues and/or rhizospheric soils of plants inhabiting extreme environments, such as the Siani, Kousséri, and Atacama Desert, and tested for plant growth promotion in crops such as tomatoes and corn. Among the beneficial capacities identified in these works are indole acetic acid (IAA) and ammonia production, P-solubilization, ACC deaminase, extracellular enzymatic and antimicrobial activities (84–88).

Both *Klebsiella* and *Brevibacillus* are easy-to-culture generalists, characterized by metabolic versatility and wide ranges of tolerance to abiotic factors, which could explain the exerted competitive exclusion during the culture on the R2A medium. The fact that these genera are easy to grow and work in laboratory-controlled conditions is probably the reason why there are so many studies on their abilities to promote plant growth (82, 84). Also, all the previously mentioned characteristics and beneficial traits of these bacteria could account for the growth promotion evidenced by our experiments with lettuce plants. Even though their abundance was not majority in the pot soils, they must have an important role in the experiment’s outcome. The lettuce plants obtained beneficial capabilities from the cultures as evidenced by statistically significant increases in all parameters used to evaluate growth. Similar to the results obtained in previous works, formulated/defined microbial consortia or isolated strains were used as biofertilizers to boost plant growth (45, 89, 90). Contrary to our approach, a direct culture was used as a bioinoculant, which has not been reported before.

Even though the R2A medium was formulated for water samples, it has been widely used to cultivate soil microorganisms, as it promotes low-growing heterotrophic bacteria (91). It has been demonstrated that this medium captures much more diversity compared to many other widely used ones (Blickfeldt, Brain Heart Infusion, Frazier, Trypticase Soy, Lysogeny Broth, and Nutrient and Yeast Extract), and its use has been promoted for the metabolomic profiling of soil bacteria (92, 93). Therefore, we believe that this culture medium and conditions promoted a competitive advantage for *Klebsiella* and *Brevibacillus* since both are generalists, versatile, and adaptable (80, 88). This, added to the fact that they are easily isolated, cultured, and managed in the laboratory, makes them ideal biofertilizer candidates.

Although the taxonomic composition of the cultures ended up being equivalent, there were differences between the rhizosphere soils of both plants; *Pseudomonas*, *Bacillus,* and *Paenisporosarcina* were the main genera of *S. foliosa*, while *Porphyromonas* and *Haloferax* dominate *D. spicata* rhizobiome, all of which were depleted by competitive exclusion during the culture stages. *Pseudomonas* has a long-lasting relationship with plants and is recurrent in desert environments. It is widely reported that it has the capability to colonize plant surfaces and inside tissues, thus promoting plant growth by suppressing pathogens and synthesizing phytohormones (94, 95). Nonetheless, some species such as *Pseudomonas syringae* is a well-known plant pathogen (96). Moreover, the *Bacillus* genus is well known for its versatility, stress tolerance, and ability to form very resistant spores. Particularly, its interactions with plants have also been widely studied, demonstrating its ability to colonize the roots through biofilm formation, stimulate growth, act as a biocontrol agent, and facilitate tolerance to abiotic stress (97, 98). This agrees with our findings due to the Yungay area conditions and reaffirms the relevance of these genera for agriculture improvement in the context of a climate change scenario. Also, bacteria from the *Paenisporosarcina* genus (previously classified as *Sporosarcina*) have been identified as Gram-positive, spore-forming, generalist, coccobacillus, and some can be psychrophilic (99). This genus has been recurrently detected and cultured from the rhizosphere of different plants in environments such as the Bolivian Altiplano and the Luoyang province in China, presenting a wide range of tolerance to temperature, drought, limited carbon sources, and even heavy metals (100, 101). Interestingly, this genus has also been reported as the second most detected endophyte of *Atriplex* spp. in the Kalahari Desert and Jornada del Muerto; this plant belongs to the Chenopodioideae subfamily along with *S. foliosa* (102).

The archaea genus *Haloferax,* characterized as a denitrifying halophile, was also detected as a majority component of *D. spicata* rhizobiome. This microorganism has also been associated with benefits for plants, particularly the production of phytohormones such as IAA that promote growth and siderophores that contribute to changing the soil physicochemical properties and mitigating stress (103–105). Additionally, we detected the genus *Porphyromonas* in high abundance, which is considered a pathogen (106). Although there are some reports of this genus in environmental samples (107), we did not find any report that associates this anaerobic bacterium with plants or any beneficial capacity.

By tracking the taxa present in the rhizosphere soils of Yungay, which persisted during culture and were detected in the pot soils after the biofertilization experiment, we identified microorganisms belonging to the genera *Klebsiella, Pseudomonas, Paenisporosarcina, Ammoniphilus resine, Enterococcus, Bacillus,* and *Paenibacillus* and the families Planococcaceae and Bacillaceae. As those mentioned above, these organisms have been described previously as having beneficial capacities for the plants with which they interact. In particular, *Enterococcus* produces different phytohormones and can promote tolerance to salt stress (108, 109). On the other hand, *Paenibacillus* has the ability to fix nitrogen and also inhibits phytopathogen nematodes (110, 111). On the other hand, the *Ammophilus* genus is very interesting because it is an oxalotrophic bacteria that can secrete organic matter hydrolases to accelerate substance degradation and promote nutrient recycling (88). Although there are no reports that associate *Ammoniphilus* with plant growth promotion, if it has been detected in rhizosphere soils (112, 113). Interestingly, two members of this genus are part of the co-occurring community, and one of them is also identified as a keystone species. Moreover, candidates *Klebsiella, Pseudomonas, Paenisporosarcina,* and a *Bacillaceae* member were also part of the co-occurring community, which agrees with the findings and implies an important role for these taxa that are worth continuing to investigate.

The other identified keystone species (*Longispora* sp., *Novibacillus thermophilus*, *Planifilum* sp., *Streptomyces* sp., *Chryseolinea* sp., and a Gemmatimonadota member) may also be targets for more mechanistic research, but we must consider that the origin of some of these could be the lettuce plants’ own microbiome (114). Finally, as the growth promotion effect was greater in the lettuce plants inoculated with the culture generated from the *S. foliosa* rhizospheric soil, we would like to point out that in this culture, 9 of the 11 identified candidates were present, while in *D. spicata,* only 5 of the 12 were detected. Furthermore, also in the *S. foliosa* cultures, *Pseudomonas* was less abundant and *Paenibacillus* was more abundant, which can also give us clues to discover which organisms may have more influence on the observed results.

### Conclusion

The relevance of this investigation is the direct use of cultures generated from the rhizospheric soil of plants thriving under the harsh conditions of the Atacama Desert hyper-arid core. Clearly, the culturable fraction of these rhizobiomes is able to transfer key traits to the crop to improve its growth and yield, which is very appealing as the use of a small group of microorganisms that are easily grown in laboratory-controlled conditions can have a significant and positive effect over an economically important crop. It is important to mention that all these experiments were carried out in the city of Antofagasta, which is under desert climate; therefore, the plants were subject to several of these conditions even though they were maintained in a shadehouse with constant watering. Also, we propose, as the next step, to isolate *Klebsiella* strains and test their plant growth-promoting effect in monoculture due to their possible role on the obtained results. Finally, we want to highlight the novelty of the work and relevance of the results obtained, this being the first report where the taxonomic composition of soil cultures is monitored over time and subcultures and for the evaluation of bacterial consortia from the Atacama Desert native plants as good biofertilizers.

## Data Availability

The whole amplicon sequencing raw data sets have been deposited at DDBJ/ENA/GenBank under the BioProject: PRJNA971922.

## SUPPLEMENTAL MATERIAL

Supplemental material spectrum.00056-24-s0001.pdf Fig. S1 to S4.

ASM does not own the copyrights to Supplemental Material that may be linked to, or accessed through, an article. The authors have granted ASM a non-exclusive, world-wide license to publish the Supplemental Material files. Please contact the corresponding author directly for reuse.
