# Supplementary material for: Rhizospheric bacteria from the Atacama Desert hyper-arid core: cultured community dynamics and plant growth promotion
Source: Microbiol Spectr. 2024 Apr 30;12(6):e00056-24. doi: 10.1128/spectrum.00056-24 (PMC11237387; doi:10.1128/spectrum.00056-24)
Supplement: Supplemental material — Fig. S1 to S4. [file spectrum.00056-24-s0001.pdf]

SUPPLEMENTARY MATERIAL

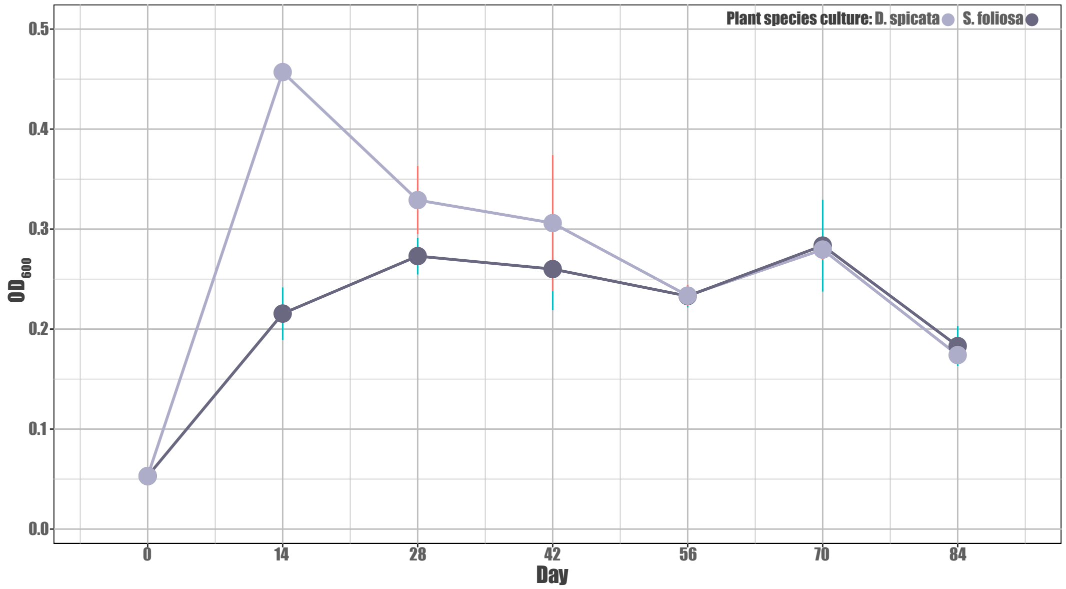

Supplementary Figure S1. Optical Density (600 nm) monitoring of *D. spicata* and *S. foliosa* rhizospheric soil cultures for 84 days. The points represent the replicates average, colored by the plant species.

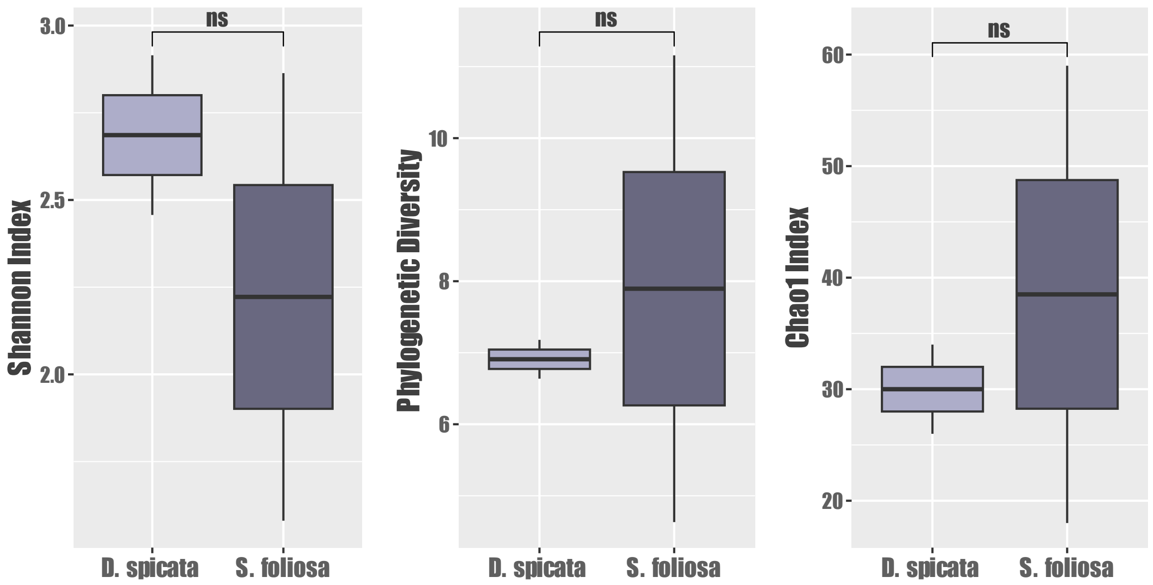

Supplementary Figure S2. Variability of alpha diversity indices for the microbial communities between both plant species rhizospheric soil.

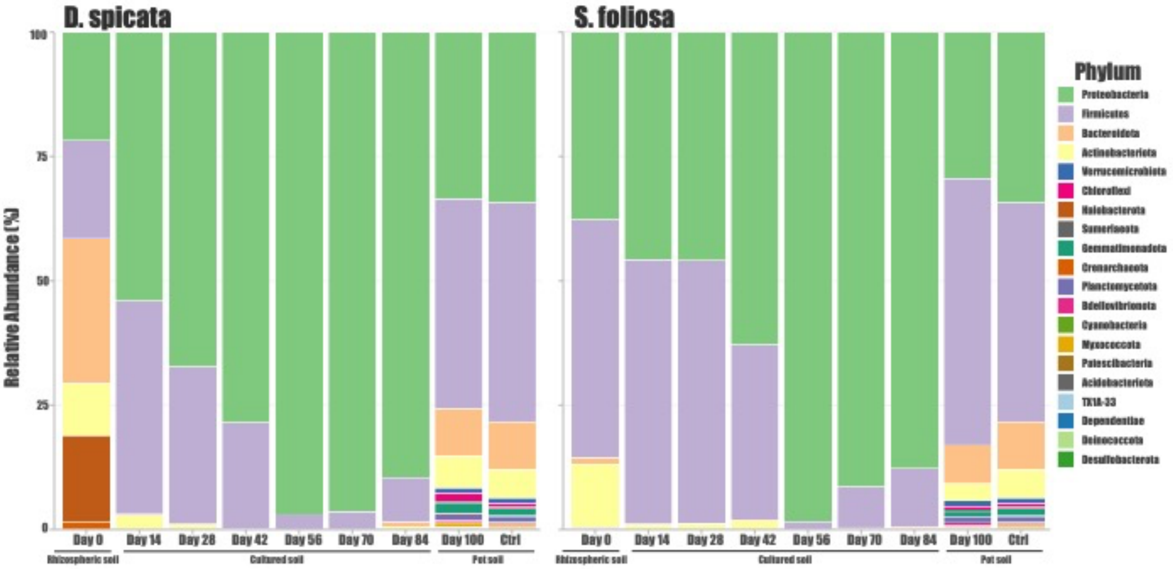

Supplementary Figure S3. Taxonomic composition dynamics through time and experimental stages. Taxa were agglomerated to display the relative abundance of the top 20 phylum ranks (code by colors).

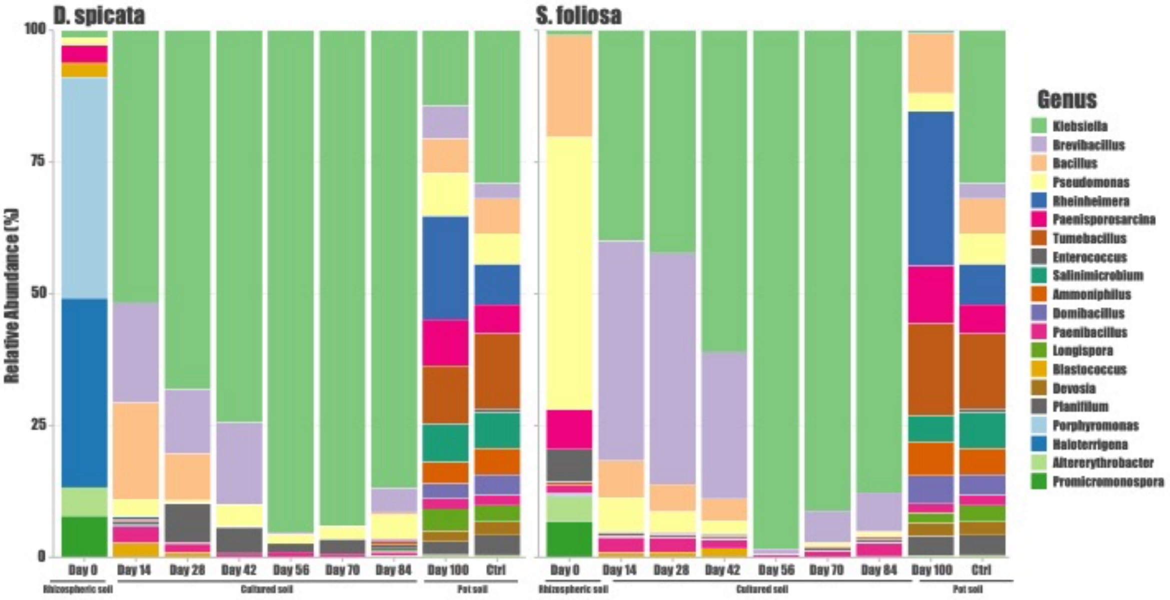

Supplementary Figure S4. Taxonomic composition dynamics through time and experimental stages. Taxa were agglomerated to display the relative abundance of the top 20 genus (code by colors).
